# A Treatise on the Practice of Medicine

**Published:** 1852-10

**Authors:** 


*A Treatise on the Practice of Medicine.* By GEORGE B. WOOD, M. D., Professor of the Theory and Practice of Medicine in the University of Pennsylvania, &c. &c. Third Edition, 2 vols. Philadelphia: Lippincott, Grambo & Co., 1852. 8vo. pp. 847 and 853, including index.

We regret that, owing to a derangement of editorial plans, a notice of this last edition of Dr. Wood's standard work has not appeared before, and our apologies for the delay are due to the author and our readers. In common with all who take an interest in American medical literature, we have felt the greatest satisfaction in the appearance of these rapidly succeeding editions of this excellent book—affording as they do, the gratifying evidence that it has taken the hold which it deserves upon the profession of our country, and is received as the national textbook upon the Practice of Medicine.

Previous notices of the earlier editions of this work, render superfluous any extended analysis of the edition just issued—"the treatise remaining unchanged in all its essential features." The modifications and alterations necessary to adapt it to the present state of medical knowledge, are, however, numerous. "Among the additions may be mentioned brief notices of several diseases, which, from their rarity or total absence in this country, or from being yet unknown as distinct affections, were not described in the former editions. Such are the relapsing fevers of Dr. Jenner, the leucocythemia of Professor Bennet, the dengue, and certain cutaneous affections, as trichosis, lupus, and pellagra." Modifications have been introduced on the subjects of inflammation, fatty degeneration, carcinoma, epidemic cholera, phthisis, hemorrhage, Bright's disease, &c. Extended reference has also been made to the important microscopic discoveries of recent times, and their bearing upon pathology.

Dr. Wood's views on the value of *cod-liver oil* in the treatment of phthisis have undergone great modifications since the publication of the last edition of his work. Upon so generally interesting a subject we believe that it will be acceptable to our readers that the opinion of such eminent authority should be quoted in extenso :—

"When the last edition of this work was published, the experience of the author had not been favorable to that remedy; but this was simply because he had not persevered with it a sufficient length of time, in the several cases in which it was employed. The oil seldom produces any very observable effect under a period of from three to six weeks; and, despairing of any good result, he had in every instance omitted it too soon. Subsequent experience has convinced him of its inestimable value. For the last three years he has used it in nearly every case of phthisis which has come under his notice either in private or hospital practice, and almost always, when it could be retained on the stomach, with either temporary or lasting benefit. His experience coincides generally with that of the writers who have testified favorably of its effects. In the worst cases, and most advanced stages, it usually improves the condition of the patient, renders him more comfortable, and postpones the fatal issue. Under less desperate circumstances, it often arrests for a time the march of the disease, giving hopes even of ultimate recovery; and, in some few instances, these hopes are justified by the result, so far as time has hitherto enabled us to judge. Given at the first appearance of the symptoms, I have frequently found it to set them aside altogether, and believe that, with the aid of other suitable remedies, it is capable of effecting permanent cures in very many instances. Its observable effects are usually to improve the digestion, to render the patient fatter and stronger, to diminish the frequency of the pulse and to check the sweats at night, to relieve the cough, and, in fine, greatly to ameliorate all the general symptoms. The patient often becomes fleshy, exchanges his paleness for a ruddy or healthy color, and feels himself nearly if not quite well. Unhappily, the physical signs do not generally undergo the same rapid improvement. If there was evidence of considerable solid deposition, this goes on to softening and the formation of a cavity; if there was a cavity at the commencement of the treatment, this not unfrequently enlarges, while others form; and, after a period of very flattering amendment, perhaps for months, perhaps for a year or more, the symptoms return, and the patient too often sinks at last. Yet it is not always so. Sometimes suspicious physical signs in the early stage entirely disappear, and cavities either remain stationary or heal. The following, so far as I am capable of judging from my own observation, and the recorded experience of others, appears to be the real value of the remedy. It does not act as a specific, and is wholly incapable of producing, by any direct influence of its own, the removal of the deposited tuberculous matter. But it invigorates digestion, improves the character of the blood, and by a peculiar power modifies the nutritive process, so as to obviate, in a greater or less degree, the tendency to the deposition of tuberculous matter. When this tendency is not very strong, and other suitable measures are made to co-operate with the oil, it appears capable of arresting the further formation of tubercles altogether. But the matter deposited must pass through its own destined changes. If small in quantity, as in the earliest stage of the disease, it may undergo the calcareous metamorphosis, and thus cease to do harm. If larger, it must soften and be discharged, leaving a cavity, which may ultimately heal if not increased by further accessions of tubercle. If abundant, it must undergo the same change, and then must necessarily prove fatal, should so much of the lung be destroyed in the process as to render the remainder insufficient to fulfil the purposes of respiration, or should the strength be inadequate to support the exhausting effects of the necessary irritation and suppuration. It is seen, therefore, that cod-liver oil though a very valuable agent, perhaps the most valuable, should be looked on only as one of the means of confirming the general health, and thus affording the best possible protection against the further progress of the malady, and enabling the system to withstand the depressing and exhausting influences necessarily exerted upon it in the elimination of the tuberculous matter. These views are certainly encouraging; and they would seem to be supported by the fact of the recent diminution of the general mortality from phthisis, as evinced by the statistical reports, in the city of Philadelphia, which can be ascribed to no other known cause than the general use of cod-liver oil. But great care must be taken to guard against the error of relying upon this alone, to the neglect of exercise, exposure to pure air, and the various other methods of invigorating the system, already referred to. A tablespoonful of the oil should be given three times a-day, and the remedy persevered in for many months, nay, interruptedly, even for years, should it continue to agree with the patient, and the disease not appear to be sooner eradicated. As the diathesis is often constitutional and inherited, it will be necessary to be always on the watch, even after the disappearance of the symptoms, in order to meet them promptly should they return. As the oil favors the production of a rich blood, it is obvious that, upon the occurrence of hæmoptysis or acute inflammation, it should be suspended until these have been subdued, and then resumed."

